# XL-DNase-seq: improved footprinting of dynamic transcription factors

**DOI:** 10.1186/s13072-019-0277-6

**Published:** 2019-06-04

**Authors:** Kyu-Seon Oh, Jisu Ha, Songjoon Baek, Myong-Hee Sung

**Affiliations:** 1 Laboratory of Molecular Biology and Immunology, National Institute on Aging, National Institutes of Health, 251 Bayview Boulevard, Baltimore, MD 21224 USA; 2 Laboratory of Receptor Biology and Gene Expression, National Cancer Institute, National Institutes of Health, 41 Library Drive, Bethesda, MD 20892 USA

**Keywords:** Transcription factor footprinting, Chromatin accessibility, High-throughput sequencing, Bioinformatics, Epigenomics, Genomic footprinting, DNase-seq, ATAC-seq, Computational genomics, Transcription factor binding motifs

## Abstract

**Background:**

As the cost of high-throughput sequencing technologies decreases, genome-wide chromatin accessibility profiling methods such as the assay of transposase-accessible chromatin using sequencing (ATAC-seq) are employed widely, with data accumulating at an unprecedented rate. However, accurate inference of protein occupancy requires higher-resolution footprinting analysis where major hurdles exist, including the sequence bias of nucleases and the short-lived chromatin binding of many transcription factors (TFs) with consequent lack of footprints.

**Results:**

Here we introduce an assay termed cross-link (XL)-DNase-seq, designed to capture chromatin interactions of dynamic TFs. Mild cross-linking improved the detection of DNase-based footprints of dynamic TFs but interfered with ATAC-based footprinting of the same TFs.

**Conclusions:**

XL-DNase-seq may help extract novel gene regulatory circuits involving previously undetectable TFs. The DNase-seq and ATAC-seq data generated in our systematic comparison of various cross-linking conditions also represent an unprecedented-scale resource derived from activated mouse macrophage-like cells which share many features of inflammatory macrophages.

**Electronic supplementary material:**

The online version of this article (10.1186/s13072-019-0277-6) contains supplementary material, which is available to authorized users.

## Introduction

Understanding of tissue-specific transcriptional regulome requires the knowledge about DNA sequence-specific chromatin interactions of transcription factors which are active in the given cell context. ChIP-seq has been widely adopted and currently the most common method of profiling the regulatory landscape of a transcription factor [16]. Related methods include ChIP-exo, ChIP-nexus, ChIA-PET, and HiChIP [7, 10, 21, 30]. However, numerous issues preclude their use in a systematic manner for a given cell type. Many in vivo cell subsets of interest or patient samples come with a limited number of cells, whereas typical ChIP protocols require 1–20 million cells. Sonication and immunoprecipitation with antibodies also need to be optimized and validated for each application of ChIP, and chromatin from different cell types often call for reoptimization of these steps. While ChIP-exo and ChIP-nexus were developed to produce precise locations of TF binding sites [10, 30], commonly used ChIP-seq methods can only localize TF binding sites with about 200 bp resolution. Another caveat with ChIP-based methods is that the antibody often recognizes one subunit of multi-protein complexes. For example, many TFs exist as heterodimers: AP-1 as c-Jun:c-Fos, NF-κB as RelA:p50; or homodimers: p50:p50, STAT1:STAT1. Since many of the subunits switch their partners in different cellular contexts, detecting one subunit does not distinguish which specific dimer species may occupy the site. Perhaps the most serious problems may arise from batch-to-batch variability of ChIP-seq, reflecting technical and biological variability from several sources including those mentioned above.

Genomic TF footprinting, an alternate method for identifying occupancy of a large number of TFs in one DNase-seq or ATAC-seq sample, had the potential to circumvent many of these issues with ChIP-based assays [4, 11, 23]. Chromatin accessibility assays rely on the ability of DNA-acting enzymes to distinguish protected sites from accessible sites in the chromatin regardless of their specific DNA sequence content. Although these methods robustly identify cell state-specific regulatory regions which are 150 bps or larger, footprinting efforts to infer transcription factor (TF) occupancy from nucleotide-level DNase cut count (or transposase insertion count in case of ATAC-seq) profiles face different challenges. Generating cut/insertion count data for footprinting of a large-size genome requires ultra-deep sequencing of DNase-seq/ATAC-seq libraries. Nevertheless, the improvement in sequencing technology and the decreasing cost have made genomic TF footprinting feasible for many laboratories with proper computational resources and expertise.

However, serious limitations have dampened early enthusiasms for using the chromatin accessibility analysis methods to identify TF occupancy in an unbiased high-throughput manner. First, the enzymes used to probe chromatin (DNase I and Tn5) in these assays were found to have non-negligible DNA sequence preferences for their reaction, which complicates the assumption that these nucleases non-specifically sample accessible nucleotides [9, 17, 34]. To address this issue, computational algorithms have been developed to take such sequence biases into account when putative footprints are called [1, 8, 26, 34]. Increasing the depth of sequencing can further mitigate this artifact, for example, with a more accurate adjustment for the enzyme-inherent sequence preferences directly from the data [9, 34].

**Fig. 1:**
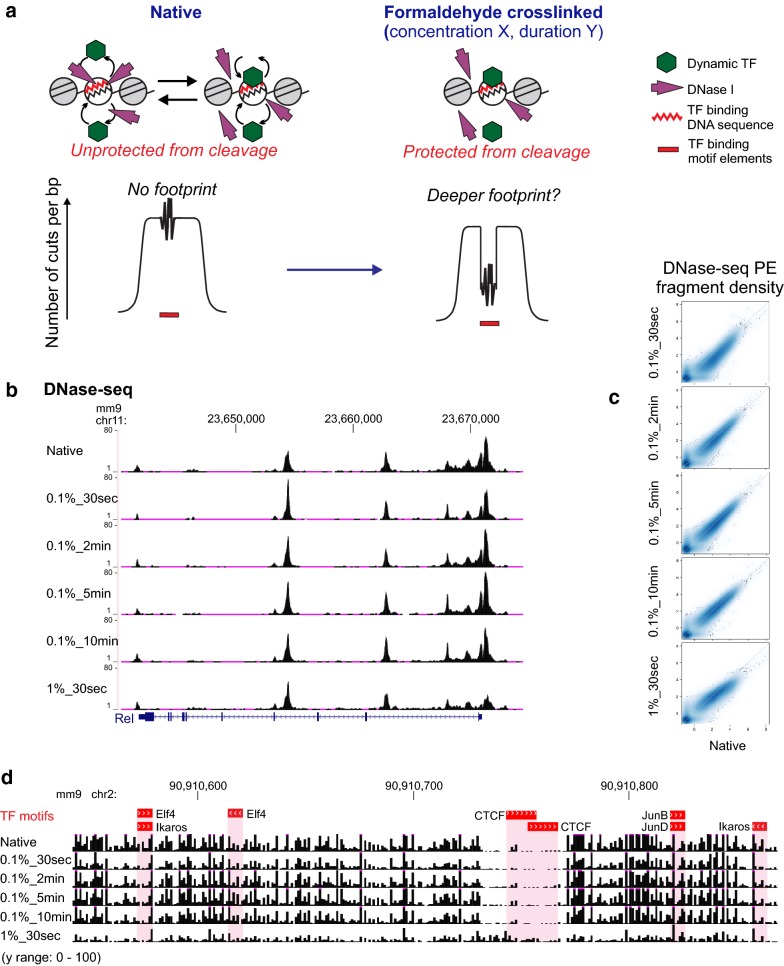
XL-DNase-seq is designed to capture short-lived binding of TFs and preserve chromatin accessibility profiles. **a** Rationale for introducing a cross-linking step prior to digestion by DNase I. **b** Near-identical DNase-seq density profiles across cross-linking conditions. XL-DNase-seq preserves the chromatin accessibility profile as observed by the native DNase-seq. The genome browser shot of the *Rel* locus shows DNase-seq signal tracks from all cross-linking conditions. **c** Fragment density normalized to 10 million mapped reads. Genome-wide comparison of DNase-seq fragment density profiles across the cross-linking conditions. **d** A browser shot of cross-linked raw cut count tracks. The top track shows the locations of TF binding motif elements obtained by FIMO. Reference genome: mm9. See also Additional file 2: Fig. S1

Another problem with genomic TF footprinting was the lack of sufficient protection of DNA from TFs whose chromatin binding is short-lived (referred to as “dynamic TFs” in this study) [9, 33, 34]. Independent live cell microscopy analyses and cross-linking analysis have established that such TFs have high exchange rates or show short chromatin residence times, which together suggest short-lived interactions of these TFs with target DNA in chromatin (see [33] for a compilation of relevant data). This is an independent and major hurdle, since it violates the basis for inferring protein occupancy through stable binding of TFs to their cognate motif elements. Consequently, little or no footprints are detectable from dynamic TFs in the cut or insertion count profiles. No solution has been proposed to address the difficulty of detecting significant footprints from such TFs. Correcting for the nuclease sequence biases produces a marginal increase in the accuracy of TF binding predictions [33, 34]. Here, we aimed to improve existing TF footprinting protocols by introducing a cross-linking step to capture highly dynamic interactions of TFs with target DNA elements in chromatin [27] (Fig. 1a). We asked whether there is an optimal cross-linking procedure which preserves and enhances the footprint depths of most TFs. We present a new footprinting protocol, termed XL(cross-link)-DNase-seq, with a mild formaldehyde cross-linking procedure that can easily be incorporated prior to the steps in the conventional DNase-seq protocols.

## Results

### Mild cross-linking prior to DNase-seq preserves chromatin accessibility and generates differential footprints

We sought to systematically assess the effects of various cross-linking procedures on the genomic footprints of dynamic TFs in the same chromatin material. For a fixed source of chromatin, we chose a cell state in which numerous TFs are directly interacting with chromatin in a cascade of gene regulatory actions. Since the chromatin sample is prepared from a cell population containing snapshots of these dynamic interactions, we reasoned that this would be a rich platform to assess changes in footprint depths of many TFs simultaneously. To this end, immortalized mouse macrophage-like RAW264.7 cells were used, where many dynamic TFs, including NF-κB and AP-1, are activated in response to bacterial products such as lipopolysaccharide (LPS). This cell context allows a large number of TFs occupying the chromatin, thereby providing an ideal platform for assessing TF footprint characteristics. We chose this cell system also because of the rich information about TF regulatory networks that the new data will help uncover in a physiologically important innate immune cell type. RAW264.7 cells have chromatin profiles which are similar to those of primary macrophages (data not shown) [24], which allows for the discovery of functionally relevant gene regulatory mechanisms [13, 18, 19, 35].

With the same chromatin material from LPS-stimulated RAW264.7, we varied the duration and the concentration of the cross-linking agent formaldehyde to determine the cross-linking parameters which may affect footprinting characteristics of dynamic TFs (Fig. 1). Based on previous reports on the dominant effect of cross-linking duration over concentration, we focused on varying the duration of formaldehyde cross-linking. A lower formaldehyde concentration of 0.1% was probed with various cross-linking durations, because a cross-linking kinetics study [27] and our pilot study indicated that 1% is a saturating concentration for cross-linking and may potentially interfere with nuclease reactions.

We have performed the modified DNase-seq, termed cross-link (XL)-DNase-seq and generated a panel of sequencing libraries. The enrichment, complexity, and quality of each library were confirmed, and all the libraries were subject to ultra-deep paired-end read sequencing (Additional file 1: Table S1). We first verified that the chromatin accessibility profile is generated independently of the mild cross-linking procedure, as observed by the reproducibility of DNase-seq fragment density across samples from various cross-linking conditions (Fig. 1b, c). This was an important first checkpoint, because excessive cross-linking may induce capture of too many non-specific factors onto the chromatin [2] and hinder sampling of chromatin by the nuclease (DNase). Generation of a DNase-seq peak relies on the ability of the enzyme to access the hypersensitive site preferentially relative to the flanking region. Our cross-linking procedure was likely mild enough to allow sufficiently differential sampling of chromatin which is reflected in the well-preserved accessibility profiles (Fig. 1b, c).

**Fig. 2:**
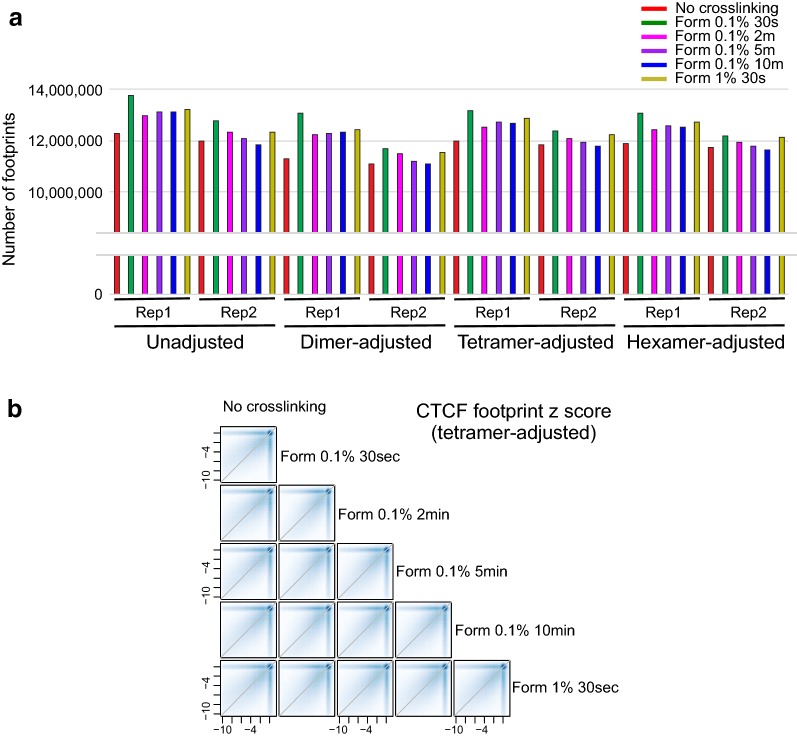
Total number of detected footprints across the cross-linking conditions. **a** Total number of FDR 1% putative footprints called by DNase2TF was averaged over five rounds of subsampling. Subsampling of reads was performed to keep the number of uniquely mapped reads the same across conditions for fair comparison (see “Methods”). **b** Footprint z score comparison across the conditions at CTCF motif sites in open chromatin. See also Additional file 2: Fig. S2

The total number of putative footprints depended on the cross-linking procedure (Figs. 1d, 2a). While the exact numbers of detected footprints differ between results from the different methods of correcting the DNase bias (dimers, tetramers, etc.), the rank order of various cross-linking samples was invariant. 0.1% 30 s XL-DNase-seq footprints produced the largest set of footprints, while the native DNase-seq produced the least number of footprints in both sets of biological replicates. Cross-linking generally preserved the footprints observed in native DNase-seq, and revealed additional footprints (Additional file 2: Fig. S2A). Since the number of footprints may change as a net result of detecting and missing true footprints, more insight can be obtained from examining specific TF footprints against a set of known binding sites. Because it is difficult to assign most footprints to specific TFs purely based on DNA sequence motifs [33], subsequent analyses focused on well-characterized TF motifs and footprints at genomic sites of their occurrences. Not only did the various XL-DNase-seq samples generate different numbers of footprints, but the footprint strengths and detectability also depended on the cross-linking procedure (Fig. 2b, Additional file 2: Fig. S2).

### XL-DNase-seq captures more TF footprints with improved accuracy

**Fig. 3:**
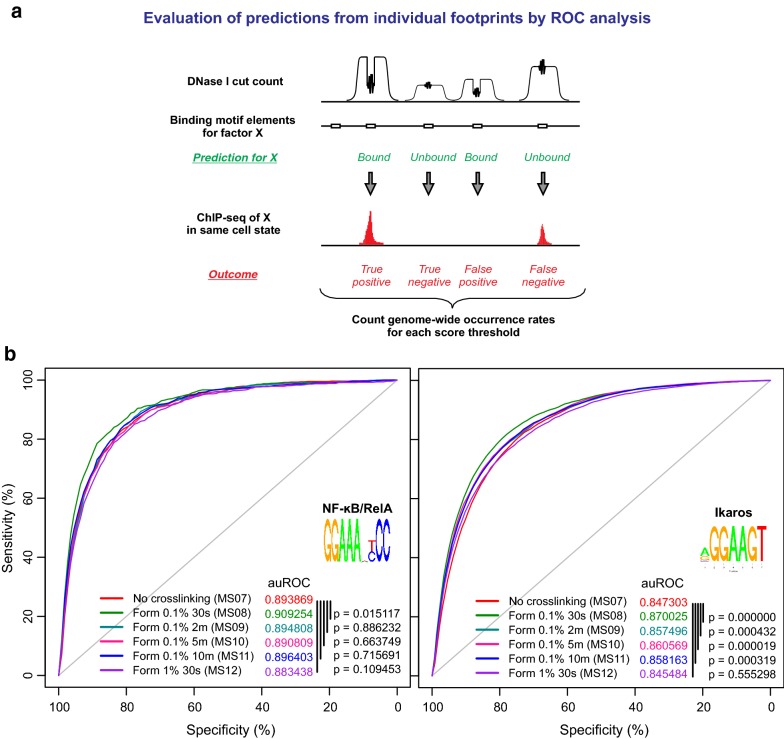
Cross-linking improves DNase-seq footprint-based prediction of TF binding. **a** Evaluation procedure in ROC analysis. **b** ROC curves for all the cross-link-DNase-seq samples are plotted using dimer-adjusted cut count data. The area under the ROC curve (auROC) values are shown along with the p values calculated by the ‘pROC’ package of R Bioconductor. Reference binding set was obtained from ChIP-seq data of LPS-activated macrophages. ROC analysis was performed for NF-κB (left: n = 99,089) and Ikaros (right: n = 165,392). See also Additional file 2: Figs. S3, S4

We analyzed the effects of cross-linking on footprinting of NF-κB/RelA, a TF which is known to have short DNA binding residence time over cognate DNA elements [3]. We assessed its footprint depths at individual κB motif sites across open chromatin in LPS-activated macrophages, using cell state-matching RelA ChIP-seq peaks as gold standard for TF binding. Receiver Operator Characteristic (ROC) analysis was performed to quantify the predictability of putative footprints from each cross-link-DNase-seq cut count data (Fig. 3a). Statistical comparison of the ROC curves revealed that NF-κB footprints obtained from 0.1% formaldehyde 30 s cross-linking (green curve) were more predictive of actual NF-κB binding in comparison to the footprints from the native DNase-seq protocol (Fig. 3b, left). This improvement was not apparent when assessed by the aggregate cut count profiles (Additional file 2: Fig. S3), underscoring the need to evaluate individual sites rather than average signals over heterogeneous sites [8, 29]. 1% Formaldehyde 30 s cross-linking (purple curve) negatively affected binding predictions. Interestingly, when we examined another TF Ikaros (which lacks microscopy data on DNA binding residence times), all XL-DNase-seq, but the 1% formaldehyde sample showed improved predictions over the native DNase-seq (Fig. 3b, right). While the observed improvement in accuracy seems small in the ROC plots, it is based on gaining several thousands of accurately predicted binding sites among 10^4^–10^5^ motif sites found within open chromatin, therefore a substantial improvement.

In all the ROC analyses, adjusting for the sequence bias of DNase cleavage did not improve the auROC values or change the overall results (Additional file 2: Fig. S4). Some studies have reported improvement of TF binding prediction from correcting the sequence bias of DNase [8], while others observed no improvement [14, 34]. The discrepant results arise probably because these studies used different computational detection methods to call putative footprints. Some detection methods rely on bias-prone cleavage signatures (shape of cut count profile) and may see significant improvement after removing the sequence bias. Our method DNase2TF does not use the cleavage signatures directly in detecting putative footprints, and therefore may have no further improvement from bias correction.

### XL-ATAC-seq does not improve TF footprinting over native ATAC-seq

**Fig. 4:**
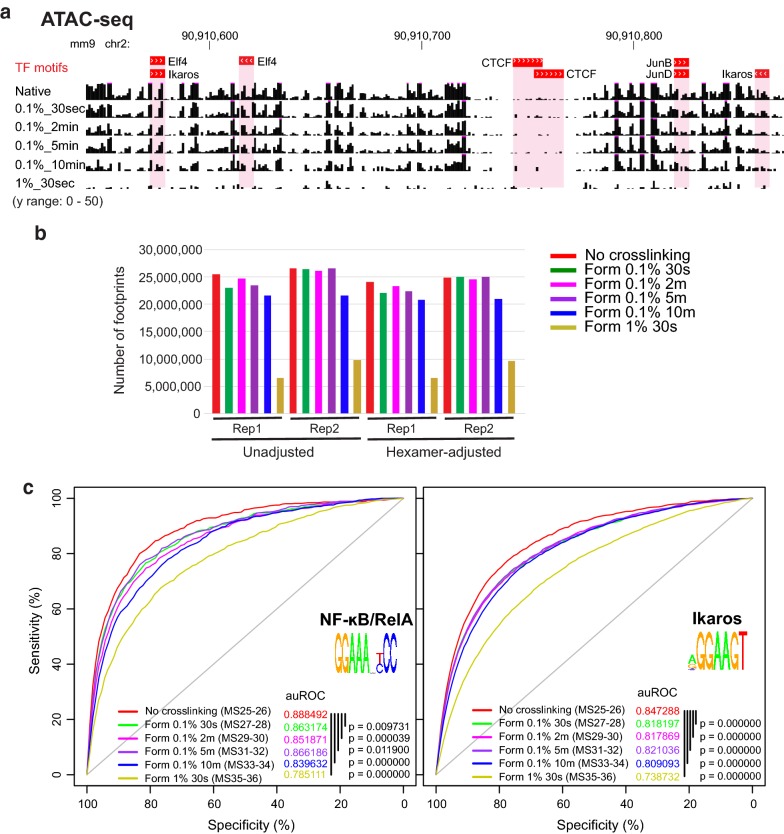
Cross-linking hinders ATAC-seq footprint-based prediction of dynamic TF binding. **a** A browser shot of cross-linked raw insertion count tracks. The top track shows the locations of TF binding motif elements obtained by FIMO. Reference genome: mm 9. **b** Total number of FDR 1% putative footprints called by ATAC2TF (DNase2TF modified for ATAC-seq) was averaged over five rounds of subsampling. Subsampling of reads was performed to keep the number of uniquely mapped reads the same across conditions for fair comparison (see “Methods”). **c** ROC curves for all the cross-link-ATAC-seq samples are plotted using unadjusted insertion count data for RelA and Ikaros. Adjusting for the sequence bias of Tn5 insertion did not improve the predictions. The area under the ROC curve (auROC) values are shown along with the p values calculated by the ‘pROC’ package of R Bioconductor. Reference binding set was obtained from RelA ChIP-seq data of primary macrophages. See also Additional file 2: Figs. S5, S6

Since the widely used ATAC-seq can also generate high-resolution footprinting data [4], we examined whether cross-linking can have similar effects as described above for DNase-seq. While the effect of short DNA binding residence times was shown in DNase-seq data, it is likely to produce similar problems for ATAC-seq. This is because dynamic TFs allow relatively long durations of dissociation from target DNA which would then be vulnerable to enzymatic attacks. We generated a panel of ATAC-seq libraries from mild formaldehyde cross-linking (XL-ATAC-seq) using the same biological material, LPS-activated RAW264.7 cells (Fig. 4a). Again, we first compared the ATAC-seq fragment density profiles obtained from all the XL-ATAC-seq samples and confirmed that the introduction of a cross-linking step does not negatively affect assaying chromatin accessibility, with only a slight reduction in signal intensity from the samples cross-linked with 1% formaldehyde (Additional file 2: Fig. S5A). However, unlike XL-DNase-seq, analysis of the insertion count data revealed that the largest number of putative footprints was detected from the native ATAC-seq samples, and the numbers declined with the duration and concentration of formaldehyde used for cross-linking (Fig. 4b). This pattern is observed regardless of whether we adjusted for the DNA sequence bias of the transposase Tn5 (used in ATAC-seq). Similarly to XL-DNase-seq, aggregate insertion count analyses did not reveal gross differences in footprint depth (Additional file 2: Fig. S6A), and footprint detectability and strength varied across XL-ATAC-seq samples (Additional file 2: Fig. S6B).

To gain a more detailed insight, we performed an ROC analysis for NF-κB/RelA and Ikaros with XL-ATAC-seq footprints, in the same manner as for XL-DNase-seq footprints. The prediction accuracy, as indicated by the shape of ROC curves and quantified by auROCs, generally declined with the duration and concentration of formaldehyde (Fig. 4a, Additional file 2: Fig. S6C). Putative footprints detected in the native ATAC-seq data (red curves) produced the most accurate predictions of NF-κB and Ikaros binding to their cognate motif elements in open chromatin. The best-performing ATAC-seq (native) nevertheless had lower auROC values compared to the XL-DNase-seq data (Additional file 2: Fig. S7A). Consistent with this observation, several studies indicated that TF footprinting based on ATAC-seq performs poorly in comparison to DNase-seq [15, 28, 29]. The cross-linking-dependent pattern of poor predictions from XL-ATAC-seq footprints was highly reproducible in technical and biological replicates. These results indicate that even the mild cross-linking conditions may interfere with the ability of Tn5 to sample the nucleotide base pairs with high insertion efficiency.

We chose ROC as our main measure of comparison, because of its wide usage for evaluating predictions from footprints and the simple representation of a random baseline as the diagonal. Another measure, precision-recall (PR) curves may also be useful especially for unbalanced binary outcome data. The PR curves generally preserved the top-performing samples (0.1% 30 s XL-DNase-seq among DNase samples and native among ATAC-seq samples), with the intermediate rankings varying somewhat over different recall ranges (Additional file 2: Fig. S7B).

### Construction of TF regulatory networks and reproducibility

**Fig. 5:**
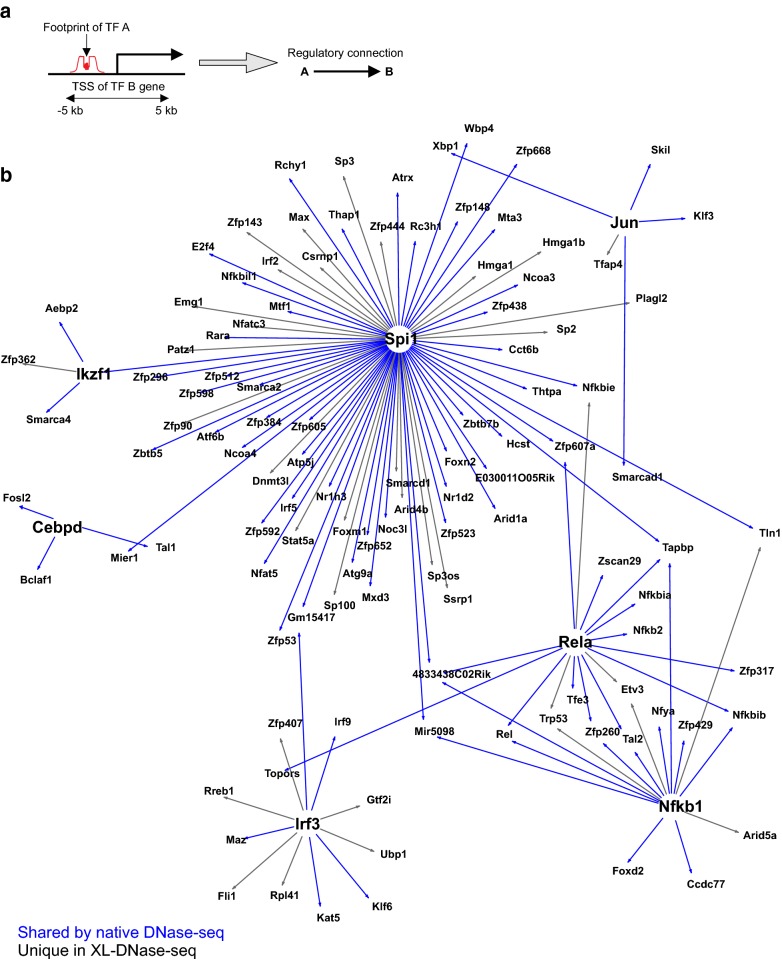
TF regulatory networks from XL-DNase-seq have reproducible connections as well as new connections. **a** De novo construction of transcriptional regulatory networks. FDR 1% footprints within 5 kb of TSSs were used to obtain regulatory relationships. **b** TF regulatory network derived from 0.1% formaldehyde 10 min XL-DNase-seq data (MS5 and MS11, merged) after correcting for the dimer bias of DNase. Regulatory connections which were also detected in the network from native DNase-seq are marked in blue. See also Additional file 2: Fig. S8

An ultimate goal of TF footprinting is to discover novel mechanisms in TF regulatory networks. A large-scale study in constructing such networks solely based on TF footprints and TF binding motif databases revealed intriguing findings [22, 32], but lack of footprints for many TFs and other technical issues raised questions about the significance of such efforts [8, 9, 33, 34]. Given the remaining technical challenges (including assignment of TF identity to motifs), we chose a conservative approach aiming to construct higher-confidence TF regulatory networks. To this end, we generated TF regulatory networks using stringent criteria on TF motifs and footprint detection thresholds. We also focused only on a select set of TFs for footprinting and limited the analysis on TF-encoding genes which have footprints of the select TFs. Each TF network was constructed by compiling putative regulatory edges (Fig. 5a, A → B, i.e., TF A regulates TF B). A regulatory edge indicates that one or more footprints of A were detected within 5 kb of transcription start site of B, from the given XL-DNase-seq sample.

Comparison of six TF networks from the cross-linking conditions revealed a substantial set of shared regulatory edges (Fig. 5b, Additional file 2: Fig. S8). Such reproducibility in regulatory relationships was surprising, given the variability observed in the footprint Z score profiles and the differential detectability of footprints across the XL-DNase-seq samples (Fig. 3, Additional file 2: Fig. S2). A closer look provided a reason for this robust consensus among the independently constructed TF networks: a shared edge is often supported by multiple redundant footprints and detection of at least one is sufficient for reproducing the edge in a network derived from a given XL-DNase-seq sample. For example, we found reproducible regulatory connections from PU.1 to many macrophage/immune-relevant genes: Ncoa3 (a.k.a. Src-3, involved in defense against bacteria) [5], Hcst (a.k.a. DAP10, which induces osteoclastogenic signaling in myeloid cells) [12], Atrx (a heterochromatin silencer), Mier1 (a HDAC-binding transcriptional corepressor), Arid1a (a.k.a. BAF250a, a component of SWI/SNF). In addition, well-known regulatory targets of RelA/NF-κB such as Nfkbia (a negative feedback gene), Rel (an immune-specific subunit of NF-κB), and Nfkb2 (an alternative dimer subunit of NF-κB) were robustly detected. RelA was also a putative regulator of Tfe3 (involved in macrophage autophagy and cytokine response, also detected as a putative target of PU.1 in multiple networks) [25] and Tal2 (a known target of PU.1) [6]. These regulatory connections were based on footprints detectable in both native and XL-DNase-seq data.

**Fig. 6:**
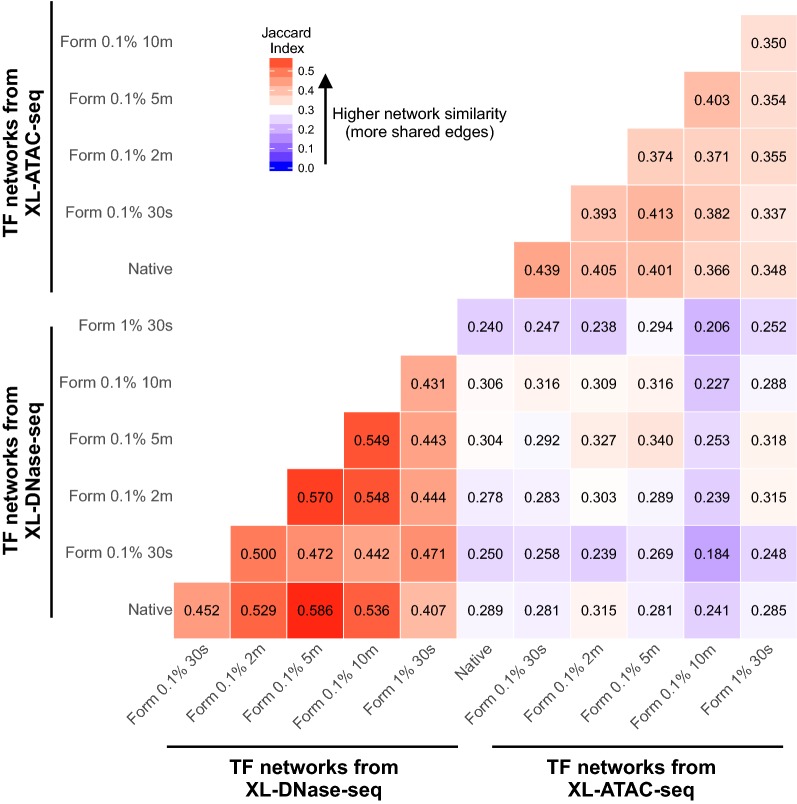
Comparison of TF regulatory networks derived from TF footprints in XL-DNase-seq and XL-ATAC-seq data. The similarity between all the pairs of TF regulatory networks was quantified using Jaccard Index (ranging from 0 to 1), and the results are represented in a heatmap. The number in each component indicates the Jaccard Index of the given pair

For an overall comparison of all the TF networks constructed separately, we generated a pairwise similarity matrix and found that XL-DNase-seq-derived networks have a stronger consensus, i.e., more regulatory edges were repeatedly detected in multiple cross-linking conditions, than XL-ATAC-seq-derived networks (Fig. 6). Despite the lower consensus among XL-ATAC-seq-derived networks, XL-ATAC-seq networks were still more similar to each other than to TF networks derived from XL-DNase-seq (Fig. 6, lower right square block). Notably, there were recurrent regulatory edges that are reproduced in multiple cross-linking conditions and even between DNase-seq and ATAC-seq protocols, which probably represent highest-confidence TF regulatory events.

Importantly, networks derived from footprints in cross-linked samples had more uniquely detected edges (Fig. 5b, black edges) than that from the native DNase-seq (Additional file 2: Fig. S8, black edges), suggesting that cross-linking helps capture novel binding events. Among the connections identified as novel regulatory relationships were: Tln1 (a.k.a. Talin, involved in mechanical responses and EMT) has a Nfkb1 footprint only from XL-DNase. Nfkbie, a known target of NF-κB in a negative feedback loop, has RelA footprint only from XL-DNase. Etv3, induced during macrophage differentiation [31] had RelA and Nfkb1 footprints only in XL-DNase. We note that Tln1, Nfkbie, and Etv3 are all likely direct targets of NF-κB/RelA because their transcripts are immediately induced by LPS treatment in macrophages [24]. Gene ontology analysis indicated that shared as well as distinct functional categories were enriched among the genes found in newly detected regulatory relationships (Additional file 2: Fig. S9). These results suggest that TF regulatory networks constructed from XL-DNase-seq contain both robust regulatory relationships and novel network wiring involving dynamic TFs whose footprints are missed in native DNase-seq.

## Discussion

Here we present a systematic comparison, designed to investigate whether introduction of a mild cross-linking step helps capture footprints of dynamic TFs in DNase-seq and ATAC-seq data. We generated technical replicates and biological replicates of LPS-activated macrophage cell line, reaching an unprecedented replicate sampling and sequencing depths. Cumulative read number from all DNase-seq and ATAC-seq replicates are 7.5 billion reads and 5.8 billion reads, respectively. Statistically significant prediction improvement was achieved in XL-DNase-seq, but cross-linking did not produce an improvement for ATAC-seq. These divergent outcomes may be related to the different enzymatic reactions employed by DNase and Tn5 and their differential efficiency in attacking cross-linked chromatin. ATAC-seq has been reported to produce TF footprints with lower binding prediction accuracy in comparison with DNase-seq by several groups using different datasets [15, 28, 29], except for one recently published study [20]. It will be interesting to re-assess ATAC-seq footprinting with the newly introduced computational approach [20]. Finally, the numerous replicates allowed us to examine reproducible features of TF footprinting, not only at the level of aggregate signals, but also at the level of individual footprints and resulting TF regulatory network wiring.

For a higher-confidence evaluation result, we have focused our analysis on a set of dynamic TFs with well-characterized sequence motifs and available ChIP-seq data in the same cell type. We found that the use of ChIP-seq from matching cell states is important, as the prediction accuracy was decreased when ChIP-seq was taken from a different time point in a stimulation time course even with the same cell type (data not shown). This and other pitfalls known to affect footprinting analysis [33] suggest that a definitive result cannot be obtained for a large number of TFs due to their uncertain motifs and limited (cell state-matching) ChIP-seq data among public databases. Therefore, we could not rely on a large-scale analysis involving hundreds of TF motifs in our attempt to accurately assess the effects of cross-linking procedures on dynamic TFs.

Previous studies have pinpointed nuclear receptors, such as GR and ER, as a special class of TFs because their chromatin binding produces “anti-footprints”, i.e., a slight enhancement of (instead of protection from) cleavage at the binding motif elements [8, 9, 34]. It would be interesting to perform our analysis using cells with ligand-activated nuclear receptors and to determine whether cross-linking enhances the depth of their footprints. However, because of the largely one-to-one relationship between ligands and nuclear receptors, a separate chromatin sample must be prepared for each nuclear receptor, making analyses of multiple nuclear receptors prohibitively expensive. Even though we could not address nuclear receptors with our cell system, these dynamic TFs may also benefit from a carefully chosen cross-linking procedure, given that they can be efficiently immobilized onto chromatin by formaldehyde in primary immune cells [36].

We have focused on interrogating cross-linking duration to identify an optimal cross-linking condition, because a previous study of cross-linking kinetics found that TF occupancy, observed by ChIP, largely depends on the cross-linking duration but is rather insensitive to the formaldehyde concentration [27]. Hence, it is somewhat surprising that we did not observe a clearly best-performing cross-linking duration which seems optimal for the TFs examined in our cross-link-DNase-seq analysis, in so far as the formaldehyde concentration is not too high. On the other hand, this result may make the assay more straightforward for other investigators to implement, because they may not need to reoptimize the duration of the 0.1% formaldehyde cross-linking for their cell types and TFs of interest. Improved footprinting of dynamic TFs with cross-link-DNase-seq, together with ever-decreasing cost of sequencing, will facilitate efforts to discover novel TF regulatory mechanisms without the need to pre-select targeting antibodies.

We have determined the effects of various cross-linking protocols on TF binding predictions based on footprints in XL-ATAC-seq or XL-DNase-seq data, in quantitative and objective terms. We demonstrate that XL-DNase-seq improves footprintability of certain TFs, while the same analysis revealed that XL-ATAC-seq fails to enhance footprinting, compared to the native protocol. These findings will critically inform investigators as they utilize the methodologies. For example, while ATAC-seq is widely popular due to its simplicity and small-scale cell yields required for the assay, XL-DNase-seq may offer unique advantages if the study calls for higher-resolution occupancy information of TFs. In addition, our dataset represents an unprecedented-scale resource for the epigenetics and immunology communities.

## Additional files

**Additional file 1.** Supplementary table and methods.

**Additional file 2.** Supplementary figures.
