# Supplementary material for: XL-DNase-seq: improved footprinting of dynamic transcription factors
Source: Epigenetics Chromatin. 2019 Jun 4;12:30. doi: 10.1186/s13072-019-0277-6 (PMC6547507; doi:10.1186/s13072-019-0277-6)
Supplement: Supplementary file 1 — Additional file 1. Supplementary table and methods. [file 13072_2019_277_MOESM1_ESM.docx]

**Table S1.** **XL-DNase-seq and XL-ATAC-seq libraries.**

| ID | Experiment type | Number of uniquely mapped non-chrM trimmed reads | Sample |
| --- | --- | --- | --- |
| MS01 | DNase-seq | 751,667,300 | B1 No Form |
| MS02 | DNase-seq | 584,007,514 | B1 0.1%, 30s |
| MS03 | DNase-seq | 401,395,864 | B1 0.1%, 2m |
| MS04 | DNase-seq | 540,918,358 | B1 0.1%, 5m |
| MS05 | DNase-seq | 436,951,654 | B1 0.1%, 10m |
| MS06 | DNase-seq | 678,801,240 | B1 1%, 30s |
| MS07 | DNase-seq | 785,019,330 | B2 No Form |
| MS08 | DNase-seq | 501,480,472 | B2 0.1%, 30s |
| MS09 | DNase-seq | 659,983,272 | B2 0.1%, 2m |
| MS10 | DNase-seq | 730,118,410 | B2 0.1%, 5m |
| MS11 | DNase-seq | 686,131,578 | B2 0.1%, 10m |
| MS12 | DNase-seq | 773,646,740 | B2 1%, 30s |
| MS13 | ATAC-seq | 489,736,980 | B1 T1 No Form |
| MS14 | ATAC-seq | 477,598,398 | B1 T2 No Form |
| MS15 | ATAC-seq | 395,923,501 | B1 T1 0.1%, 30s |
| MS16 | ATAC-seq | 437,580,559 | B1 T2 0.1%, 30s |
| MS17 | ATAC-seq | 363,325,154 | B1 T1 0.1%, 2m |
| MS18 | ATAC-seq | 365,699,004 | B1 T2 0.1%, 2m |
| MS19 | ATAC-seq | 363,751,016 | B1 T1 0.1%, 5m |
| MS20 | ATAC-seq | 412,932,480 | B1 T2 0.1%, 5m |
| MS21 | ATAC-seq | 333,092,047 | B1 T1 0.1%, 10m |
| MS22 | ATAC-seq | 349,709,062 | B1 T2 0.1%, 10m |
| MS23 | ATAC-seq | 247,225,575 | B1 T1 1%, 30s |
| MS24 | ATAC-seq | 236,122,422 | B1 T2 1%, 30s |
| MS25 | ATAC-seq | 471,297,853 | B2 T1 No Form |
| MS26 | ATAC-seq | 482,120,994 | B2 T2 No Form |
| MS27 | ATAC-seq | 501,802,003 | B2 T1 0.1%, 30s |
| MS28 | ATAC-seq | 543,979,839 | B2 T2 0.1%, 30s |
| MS29 | ATAC-seq | 429,784,200 | B2 T1 0.1%, 2m |
| MS30 | ATAC-seq | 400,608,268 | B2 T2 0.1%, 2m |
| MS31 | ATAC-seq | 403,218,226 | B2 T1 0.1%, 5m |
| MS32 | ATAC-seq | 522,796,658 | B2 T2 0.1%, 5m |
| MS33 | ATAC-seq | 421,172,085 | B2 T1 0.1%, 10m |
| MS34 | ATAC-seq | 390,265,918 | B2 T2 0.1%, 10m |
| MS35 | ATAC-seq | 328,802,586 | B2 T1 1%, 30s |
| MS36 | ATAC-seq | 333,298,431 | B2 T2 1%, 30s |

Sample description column indicates the concentration and the duration of Formaldehydie treatment for crosslinking. “Bi Tj” denote the library sample from biological replicate i and technical replicate j. “Form”, Formaldehyde. All reads were mapped to the reference genome mm9.

**Methods**

**Contact for reagent and resource sharing**

Further information and requests for resources and reagents should be directed to and will be fulfilled by the Lead Contact, Myong-Hee Sung (sungm@mail.nih.gov).

**Experimental model and subject details**

**Cell culture**

RAW264.7 cells were grown in Dulbecco’s Modified Eagle medium (DMEM; cat. no. 11995-065, Gibco), 10% fetal bovine serum (FBS; cat. no. 100-500, Gemini) at 37°C and 5% CO2. Cells were treated with 10 ng/ml LPS (cat. no. ALX-581-008-L002, Alexis Biochemicals, Salmonella minnesota R595 TLRgrade,) for 8 hours.

**Crosslink (XL)-DNase-seq**

Prior to harvest, different crosslinking conditions were used. 5x10^6^ cells (per condition) were crosslinked with either 0.1% or 1% formaldehyde at room temperature for various times (30 sec to 10 min) then quenched with 1M glycine. Nuclear extraction, permeabilization and DNase I (Sigma) digestion were performed as previously described [1-3] with slight modifications. Crosslinked cells were pelleted followed by nuclei isolation with Buffer A (15 mM Tris-Cl (pH 8), 15 mM NaCl, 60 mM KCl, 1 mM EDTA (pH 8), 0.5 mM EGTA (pH 8), 0.04% NP-40, 0.5 mM Spermidine). Chromatin from 5 million cells were digested with 60 U/ml or 80 U/ml DNase I (Sigma) for 3 min at 37 ºC followed by termination of the reaction with an equal volume of Stop buffer (50 mM Tris-Cl (pH 8), 100 mM NaCl, 0.1% SDS, 100 mM EDTA (pH 8)) at 55 ºC for 10 min, then incubated overnight at 65 ºC for formaldehyde reverse crosslinking. Next day, 100 ug/ml additional proteinase K was added and incubated at 55 ºC for 1 hour. DNA fragments were isolated by phenol-chloroform extraction and enriched by size selection over sucrose gradient. DNA fragments between 100 bp and 500 bp were precipitated and dissolved in nuclease free H2O (Ambion). Two biological replicate samples per treatment group were prepared separately for high throughput sequencing. Sequencing libraries were generated using Illumina TruSeq protocol and subject to 76 bp paired-end sequencing on Illumina NextSeq. Sample quality and library complexity were checked using an established pipeline at the NCI Center for Cancer Research Sequencing Facility.

**XL-ATAC-seq**

Assay for Transposase Accessible Chromatin (ATAC-seq) was performed as developed by Buenrostro et al. [4] with some modifications. After harvesting, 10 million cells were aliquoted into 50,000 cells each to test different crosslinking conditions as with DNase-seq. 5x10^4^ cells (per condition) were crosslinked with either 0.1% or 1% formaldehyde at room temperature for various times (30 sec to 10 min) then quenched with 1M glycine. After crosslinking, cells were pelleted at 500 × g for 5 min, followed by a wash using 50 μl of cold 1x PBS and centrifugation at 500 × g for 5 min. Cells were lysed using cold lysis buffer (10 mM Tris-Cl, pH 7.4, 10 mM NaCl, 3 mM MgCl_2_ and 0.1% IGEPAL CA-630). Immediately after lysis, nuclei were spun at 500 × g for 20 min at 4°C. Nuclei were re-suspended in the 50 µl transposase reaction mix containing 2.5 µl of Tn5 transposase, 25 µl of TD buffer and 22.5 μl of nuclease free water (Nextera Sample preparation kit from Illumina). Then 10 µl of clean up buffer (900mM NaCl, 300mM EDTA), 4 µl of 5% SDS and 4.5 µl of Proteinase K (NEB) were added and incubated for 2 hours at 65°C for reversal of formaldehyde crosslinks. The transposition reaction was incubated for 30 min at 37 °C. Tagmented DNA was purified using a Qiagen Minelute kit. Sequencing libraries were prepared following the ATAC-seq protocol^4^ and subject to 76 bp paired-end sequencing on Illumina NextSeq at the NCI Center for Cancer Research Sequencing Facility. All XL-ATAC-seq have been performed in two biological replicates and each biological replicate was performed in two technical replicates.

**Filtering and normalization of XL-DNase-seq and XL-ATAC-seq data**

All the XL-DNase-seq and XL-ATAC-seq libraries were deeply sequenced at the NCI Sequencing Facility, Frederick National Laboratory for Cancer Research. The raw reads were filtered for quality and uniquely mapped reads were retained for further analysis. Reads mapping to mitochondrial DNA (chrM) in BAM files were excluded. Samtools (version 1.5) was used with the option “-q 10” [5]. To normalize all the data files for a fixed number of mapped reads and conduct a fair downstream comparison across crosslinking conditions, we performed down-sampling of the bam files to match the lowest number of read ends among the BAM files in each dataset. For XL-DNase-seq, normalization was applied to all 12 BAM files; for XL-ATAC-seq, the four samples from 1% Formaldehyde crosslinking produced very low read counts after filtering (due to predominant chrM reads), and normalization was applied to each biological replicate set separately after excluding these four samples. Subsampling (without replacement) was repeated five times to mitigate random sampling effects, and five subsampled datasets were generated using the reservoir random sampling algorithm [6]. These sequencing depth-normalized data files were used for the ROC analyses and aggregate plots, while full data files were used for footprint Z score scatter plots and TF regulatory network construction.

**Detection of putative footprints using DNase2TF**

First, we generated a common set of open chromatin regions as follows. DNase2hotspots was applied to detect DNase I hypersensitive sites (DHSs) in each sequencing library data file [7]. The detection threshold was set with FDR 1%. The Z-score threshold for initial filtering of hotspot candidates was 2. The detected hotspot regions from all libraries were merged to create a combined (union) set of hotspots by Bedtools (ver. 2.27.1) [8]. This was used as unified input for DNase2TF, providing a fixed footprint search space.

We applied DNase2TF to each sequencing sample data using the Z-score threshold of 3 and 100% FDR to obtain all the footprint candidates without filtering [9]. Correction of enzyme sequence bias was conducted by using the “calcDFT” utility of DNase2TF. We estimated the frequency of k-mers (dimers, tetramers, or hexamers) from the cut count data of native DNase (or ATAC)-seq replicates. In other words, for XL-DNase-seq, MS1 and MS7 bam files were merged and used as input to calcDFT. This reference frequency table was used to adjust cut count data for the enzyme sequence bias in DNase2TF runs on all the samples. We did not use crosslinked samples for estimating k-mer frequencies, because it is not known whether the crosslinking may interfere with the full sampling activity of DNase or Tn5. By fixing the search space and the k-mer frequency estimates across the XL-DNase-seq samples, we focused on the observed cut count data in our comparative footprinting analyses. Otherwise, the prediction outcomes might also be influenced by different search spaces or the accuracy of k-mer frequencies.

XL-ATAC-seq data was handled similarly, except that the starting positions of reads were shifted according to Buenrostro et al. [4]. Briefly, one combined set of hotspots was obtained from taking the union of all the hotspots detected in 24 individual XL-ATAC-seq samples. For bias correction, we merged the four native ATAC-seq data (MS13, MS14, MS25, MS26) into one bam file and used it as input for calc2DFT to generate the reference bias frequency table for hexamers.

**TF binding motifs and ChIP-seq data**

For NF-κB/RelA (GH1398) and Ikaros (GH1405), we used our ChIP-seq data from mouse bone marrow-derived macrophages activated with LPS for 8 hours [3, 10]. Additional macrophage ChIP-seq datasets from the Smale laboratory were downloaded from the NCBI Gene Expression Omnibus (GEO) database:

<https://www.ncbi.nlm.nih.gov/geo/query/acc.cgi?acc=GSE99895>

GSM2663837 CEBPb

GSM2663842 CEBPd

GSM2663847 Fos

GSM2663852 JunB

GSM2663857 JunD

GSM2663862 ATF3

GSM2663867 CREB

GSM2663885 Input

Elf4 ChIP-seq data [11] was downloaded also from GEO:

<https://www.ncbi.nlm.nih.gov/geo/query/acc.cgi?acc=GSE88699>

ChIP-seq data were aligned using STAR to mm9 [12]. We used DNase2hotspots to call TF binding sites using the ChIP-seq data and matching input DNA as negative control.

MEME suite (version 4.12.0) was used for motif analyses [13]. With TF binding regions (detected in the ChIP-seq data using DNase2Hotspots) as input, we ran DREME to identify enriched motifs [14]. To assign TFs to discovered motifs, we used position weight matrices (PWMs) from Cis-BP (http://cisbp.ccbr.utoronto.ca/, downloaded on December 6^th^, 2017) and JASPAR (http://jaspar.genereg.net/, downloaded on January 5^th^, 2018). For CTCF, we used the motif M6125_1.02 from Cis-BP. Selected PWMs were input to FIMO for scanning the mm9 genome sequence [15]. FIMO was run with a p-value cutoff of 10^-4^, using “--max-stored-scores 1500000 --thresh 0.0001”.

**Receiver Operator Characteristic (ROC) analysis**

The ROC evaluation of TF binding predictions using footprints was performed as previously described [9]. The Z-scores from DNase2TF results were used for thresholds of varying stringency. A putative footprint was assigned to a TF if it overlaps a TF FIMO motif site by one nucleotide or more. The R package “pROC” was used to calculate p values for a pairwise comparison of two ROC curves [16]. auROC values were averaged over the 5 independent sequencing depth-normalized subsamples. A Precision-Recall (PR) analysis was also done using the pROC package. Plots of the PR curves were generated by the “coords” function.

**Footprint-derived TF regulatory network analysis**

Footprints were called by DNase2TF with a Z-score threshold of zero and at 1% FDR. DNase-seq cut count data were adjusted for dimer biases of DNase I. ATAC-seq data were not adjusted. We did not repeat the network analysis using data adjusted for tetramer or hexamer biases, because ROC analysis and other assessments showed no improvement of TF footprinting from these adjustments.

Experimentally evaluated TF binding motifs were selected from Cis-BP [17] and JASPAR [18] as follows:

Jun: M0311_1.02 (Cis-BP)

Irf3: M1263_1.02 (Cis-BP)

Spi1: MA0080.3 (JASPAR)

Cebpd: M0274_1.02 (Cis-BP)

Nfkb1: MA0105.1 (JASPAR)

Rela: M6137_1.02 (Cis-BP)

Ikzf1: M3450_1.02 (Cis-BP)

Proximal regulatory sites were defined to be within 5 kb of annotated gene transcription start sites, were extracted for further analysis. The extracted sequences were from the “Mmusculus.UCSC.mm9.knownGene” build in Bioconductor [19]. Motif sites were identified by searching the TSS-proximal sequences with FIMO using a p value threshold of 10^-5^. After detection of 3bp overlapped at least, TF binding sites by FIMO with footprint regions, the relationships were established “regulator” and “regulated” with pair of TF binding sites and their detected proximal regulatory sequences of genes. Filtered out non-transcription factor genes and annotation with TF genes by using Bedtools “intersect” followed the transcription factor genes list from FANTOM5 of RIKEN (<http://fantom.gsc.riken.jp/5/sstar/Browse_Transcription_Factors_mm9>). Visualization of the constructed networks was produced by using the R package “igraph” [20]. Jaccard Index was computed as a measure of similarity between networks.

**Gene ontology analysis**

Terminal node genes of edges were provided as input to the “GOstats” Bioconductor package [21]. The function “hyperGTest” was used to perform the hypergeometric test of statistically significant enrichment for each category with respect to the genome-wide annotation database for mouse, org.Mm.eg.db (Carson M, 2018, R package version 3.7.0). The definition of enrichment score is defined to be -log_10_(p-value from the hypergeometric test).

**Data and software availability**

All data have been deposited in the European Nucleotide Archive at the links, <https://www.ebi.ac.uk/ena/data/view/PRJEB23174> (MS1-12) and <https://www.ebi.ac.uk/ena/data/view/PRJEB22841> (MS13-36).

**Supplemental Figures**

**Figure S1. Comparison of chromatin accessibility profiles from XL-DNase-seq samples.**

**Figure S2.** **Comparison of XL-DNase-seq samples at the TF footprinting resolution.**

**Figure S3.** **Average cut count log ratio profiles across the crosslinking conditions over TF motifs.**

**Figure S4. Summary of ROC analysis for TFs with available macrophage ChIP-seq data.**

**Figure S5.** **Comparison of XL-ATAC-seq samples.**

**Figure S6. TF footprinting with XL-ATAC-seq.**

**Figure S7. Comparison of TF binding predictions from XL-DNase-seq and XL-ATAC-seq.**

**Figure S8. TF regulatory network constructed by footprints from native DNase-seq.**

**Figure S9. Genomic features and ontology analysis of footprints from XL-DNase-seq.**

**References**

1. John S, Sabo PJ, Thurman RE, Sung MH, Biddie SC, Johnson TA, Hager GL, Stamatoyannopoulos JA: **Chromatin accessibility pre-determines glucocorticoid receptor binding patterns**. *Nat Genet* 2011, **43**(3):264-268.

2. Morris SA, Baek S, Sung MH, John S, Wiench M, Johnson TA, Schiltz RL, Hager GL: **Overlapping chromatin-remodeling systems collaborate genome wide at dynamic chromatin transitions**. *Nat Struct Mol Biol* 2014, **21**(1):73-81.

3. Oh KS, Patel H, Gottschalk RA, Lee WS, Baek S, Fraser IDC, Hager GL, Sung MH: **Anti-Inflammatory Chromatinscape Suggests Alternative Mechanisms of Glucocorticoid Receptor Action**. *Immunity* 2017, **47**(2):298-309 e295.

4. Buenrostro JD, Giresi PG, Zaba LC, Chang HY, Greenleaf WJ: **Transposition of native chromatin for fast and sensitive epigenomic profiling of open chromatin, DNA-binding proteins and nucleosome position**. *Nat Methods* 2013, **10**(12):1213-1218.

5. Li H, Handsaker B, Wysoker A, Fennell T, Ruan J, Homer N, Marth G, Abecasis G, Durbin R, Genome Project Data Processing S: **The Sequence Alignment/Map format and SAMtools**. *Bioinformatics* 2009, **25**(16):2078-2079.

6. Vitter JS: **Random Sampling with a Reservoir**. *Acm T Math Software* 1985, **11**(1):37-57.

7. Baek S, Sung MH, Hager GL: **Quantitative analysis of genome-wide chromatin remodeling**. *Methods Mol Biol* 2012, **833**:433-441.

8. Quinlan AR, Hall IM: **BEDTools: a flexible suite of utilities for comparing genomic features**. *Bioinformatics* 2010, **26**(6):841-842.

9. Sung MH, Guertin MJ, Baek S, Hager GL: **DNase footprint signatures are dictated by factor dynamics and DNA sequence**. *Mol Cell* 2014, **56**(2):275-285.

10. Oh KS, Gottschalk RA, Lounsbury NW, Sun J, Dorrington MG, Baek S, Sun G, Wang Z, Krauss KS, Milner JD *et al*: **Dual Roles for Ikaros in Regulation of Macrophage Chromatin State and Inflammatory Gene Expression**. *J Immunol* 2018, **201**(2):757-771.

11. Curina A, Termanini A, Barozzi I, Prosperini E, Simonatto M, Polletti S, Silvola A, Soldi M, Austenaa L, Bonaldi T *et al*: **High constitutive activity of a broad panel of housekeeping and tissue-specific cis-regulatory elements depends on a subset of ETS proteins**. *Genes Dev* 2017, **31**(4):399-412.

12. Dobin A, Davis CA, Schlesinger F, Drenkow J, Zaleski C, Jha S, Batut P, Chaisson M, Gingeras TR: **STAR: ultrafast universal RNA-seq aligner**. *Bioinformatics* 2013, **29**(1):15-21.

13. Bailey TL, Elkan C: **Fitting a mixture model by expectation maximization to discover motifs in biopolymers**. *Proc Int Conf Intell Syst Mol Biol* 1994, **2**:28-36.

14. Bailey TL: **DREME: motif discovery in transcription factor ChIP-seq data**. *Bioinformatics* 2011, **27**(12):1653-1659.

15. Grant CE, Bailey TL, Noble WS: **FIMO: scanning for occurrences of a given motif**. *Bioinformatics* 2011, **27**(7):1017-1018.

16. Robin X, Turck N, Hainard A, Tiberti N, Lisacek F, Sanchez JC, Muller M: **pROC: an open-source package for R and S+ to analyze and compare ROC curves**. *BMC Bioinformatics* 2011, **12**:77.

17. Weirauch MT, Yang A, Albu M, Cote AG, Montenegro-Montero A, Drewe P, Najafabadi HS, Lambert SA, Mann I, Cook K *et al*: **Determination and inference of eukaryotic transcription factor sequence specificity**. *Cell* 2014, **158**(6):1431-1443.

18. Khan A, Fornes O, Stigliani A, Gheorghe M, Castro-Mondragon JA, van der Lee R, Bessy A, Cheneby J, Kulkarni SR, Tan G *et al*: **JASPAR 2018: update of the open-access database of transcription factor binding profiles and its web framework**. *Nucleic Acids Res* 2018, **46**(D1):D260-D266.

19. Huber W, Carey VJ, Gentleman R, Anders S, Carlson M, Carvalho BS, Bravo HC, Davis S, Gatto L, Girke T *et al*: **Orchestrating high-throughput genomic analysis with Bioconductor**. *Nat Methods* 2015, **12**(2):115-121.

20. Csardi GN, T.: **The igraph software package for complex network research**. *InterJournal, Complex Systems* 2006, **1695**:<http://igraph.org>.

21. Falcon S, Gentleman R: **Using GOstats to test gene lists for GO term association**. *Bioinformatics* 2007, **23**(2):257-258.
