# Supplementary material for: XL-DNase-seq: improved footprinting of dynamic transcription factors
Source: Epigenetics Chromatin. 2019 Jun 4;12:30. doi: 10.1186/s13072-019-0277-6 (PMC6547507; doi:10.1186/s13072-019-0277-6)
Supplement: Supplementary file 2 — Additional file 2. Supplementary figures. [file 13072_2019_277_MOESM2_ESM.pdf]

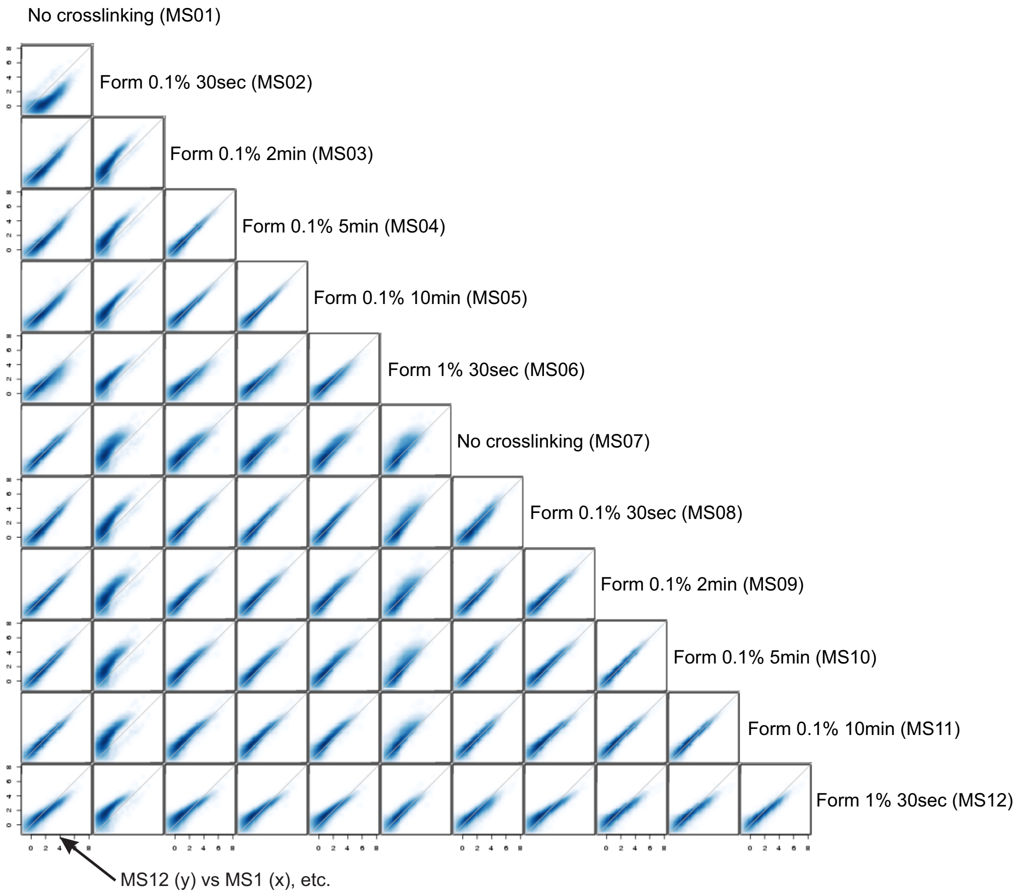

Figure S1. Comparison of chromatin accessibility profiles from XL-DNase-seq samples (related to Figure 1). Reproducibility of fragment density at all open chromatin sites (combined DNase-seq hotspots obtained by DNase2Hotspots) across the crosslinking conditions. Fragment density was averaged over each DNase hotspot. Scatter plots were generated with density estimation to visualize regions overpopulated with data points.

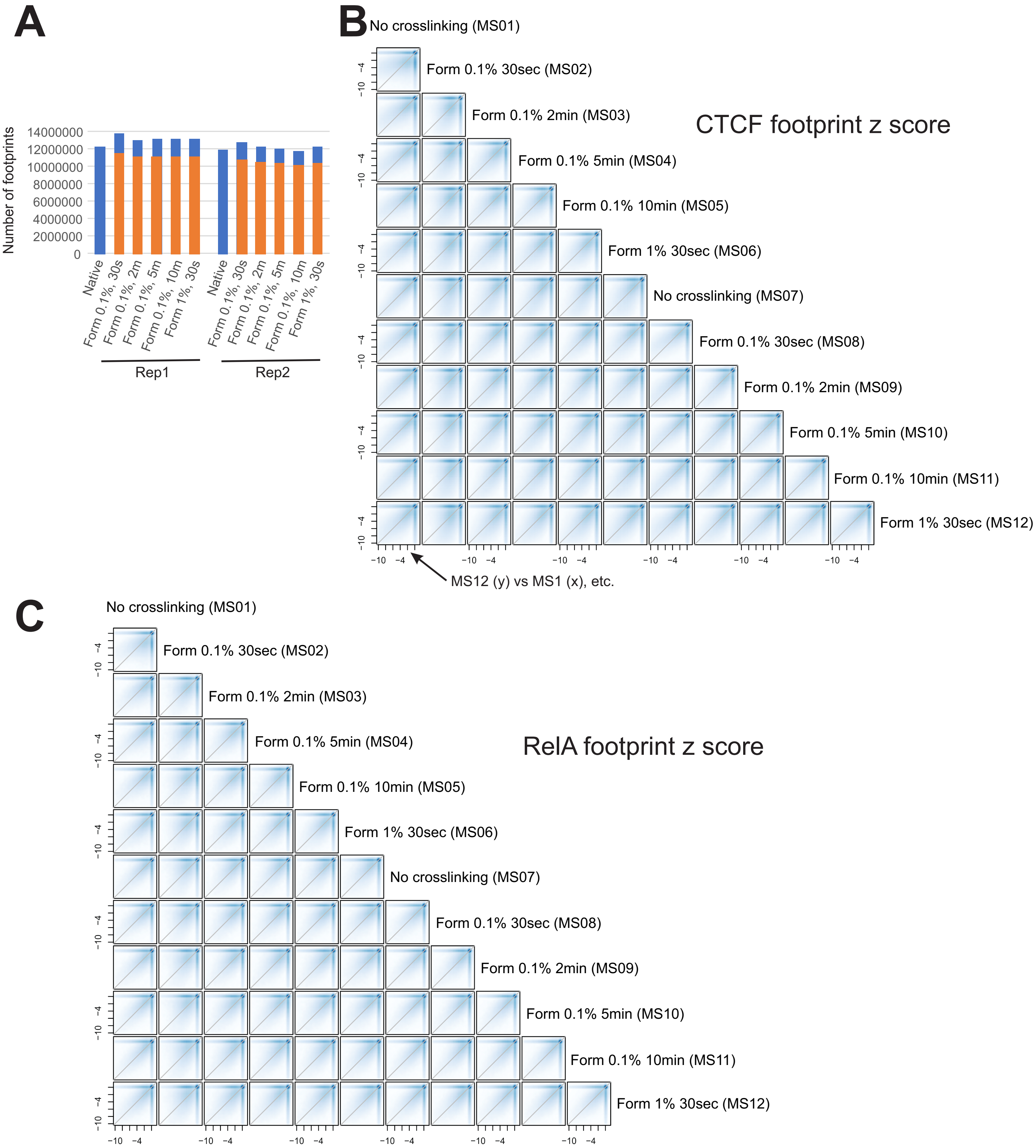

Figure S2. Comparison of XL-DNase-seq samples at the TF footprinting resolution (related to Figure 2). Subsampling of uniquely mapped reads was performed to normalize data for sequencing depth. DNase2TF was used to call putative footprints. (A) The total number of footprints from bias-unadjusted data. The orange bars indicate those overlapping footprints from the native DNase-seq. Bias correction produced similar results. (B-C) TF footprints detected after adjusting for the tetramer bias of DNase. Scatter plots were generated with density estimation to visualize regions overpopulated with data points. (B) Comparison of Z scores of FDR 1% CTCF footprints within open chromatin across the samples. (C) Comparison of Z scores of FDR 1% RelA footprints across the samples.

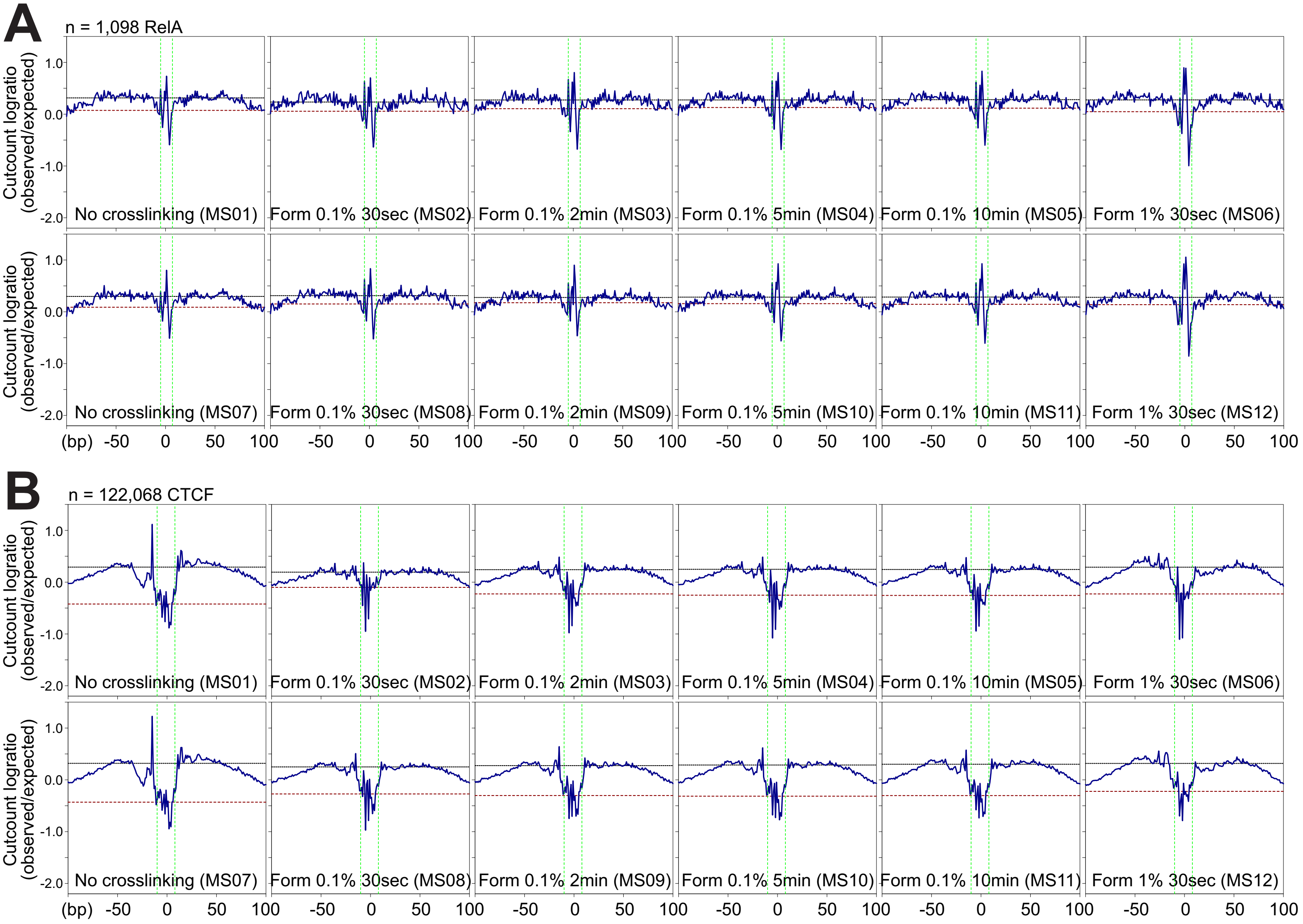

Figure S3. Average cut count log ratio profiles across the crosslinking conditions over TF motifs (related to Figure 3). Log ratios of observed cut count over expected, based on hexamer frequencies, were averaged over (A) RelA motif sites within RelA ChIP-seq peaks and (B) CTCF motif sites within DHSs (see Methods) across the crosslinking conditions. The green vertical dashed lines show the span of the motif. The black horizontal lines indicate flanking cut levels; the red dashed lines mark the average cut levels at the motif. n, total number of motif sites.

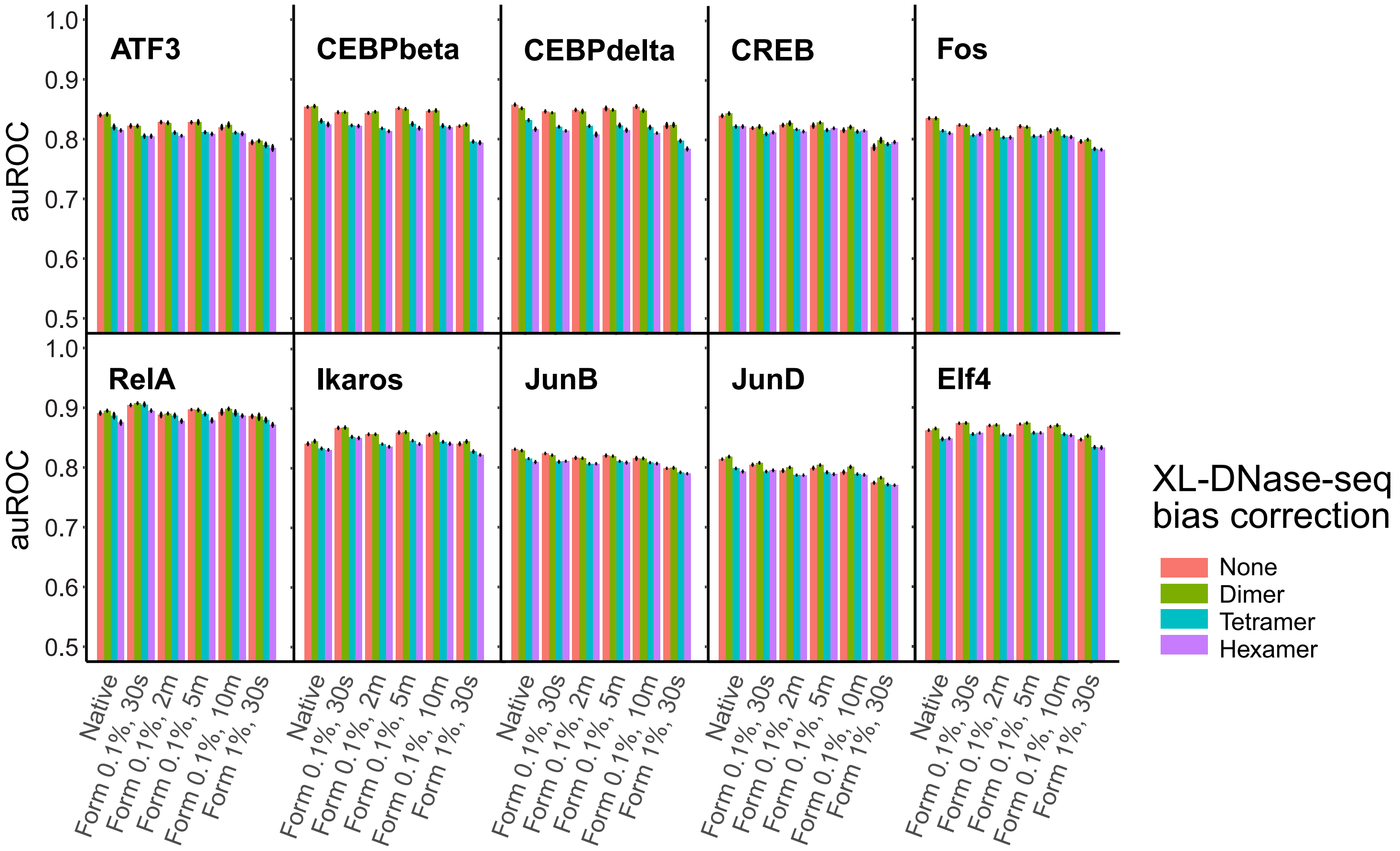

Figure S4. Summary of ROC analysis for TFs with available macrophage ChIP-seq data (related to Figure 3).

Area under the ROC curve was calculated in five rounds of subsampling the raw data. Error bars, s.d. Footprints were called by DNase2TF after correcting the cut count data for DNA sequence bias of DNase as indicated. Only RelA and Ikaros ChIP-seq data were generated from matching LPS treatment duration, and other TFs' ChIP-seq were from LPS treatment durations different from the 8h used for our DNase-seq data.

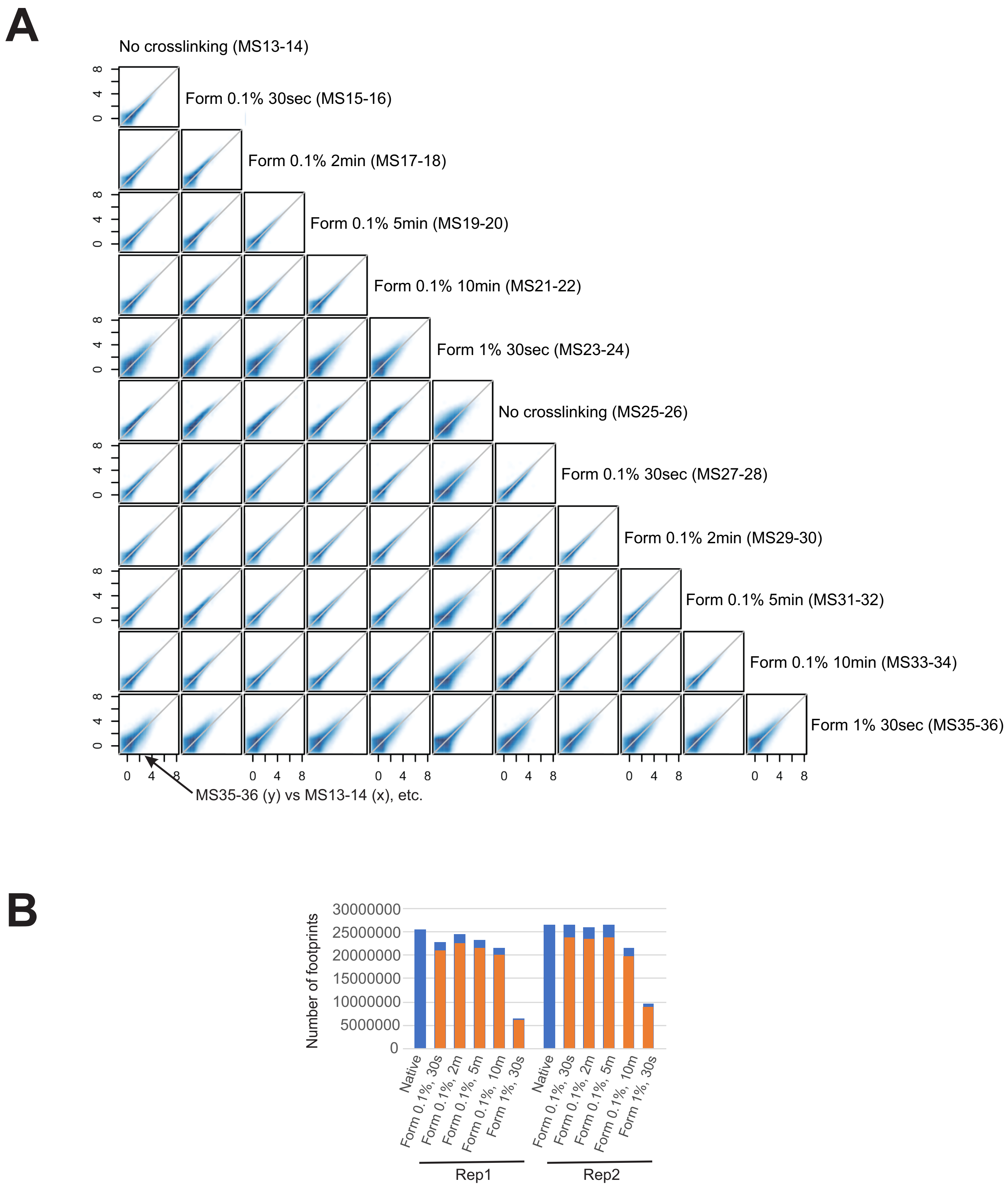

Figure S5. Comparison of XL-ATAC-seq samples (related to Figure 4).
(A) Comparison of chromatin accessibility profiles from XL-ATAC-seq at all open chromatin sites (combined ATAC-seq hotspots obtained by DNase2Hotspots) across the crosslinking conditions. Fragment density was averaged over each ATAC-seq hotspot. Scatter plots were generated with density estimation to visualize regions overpopulated with data points. (B) The total number of footprints from bias-unadjusted data. The orange bars indicate those overlapping footprints from the native ATAC-seq. Bias correction produced similar results.

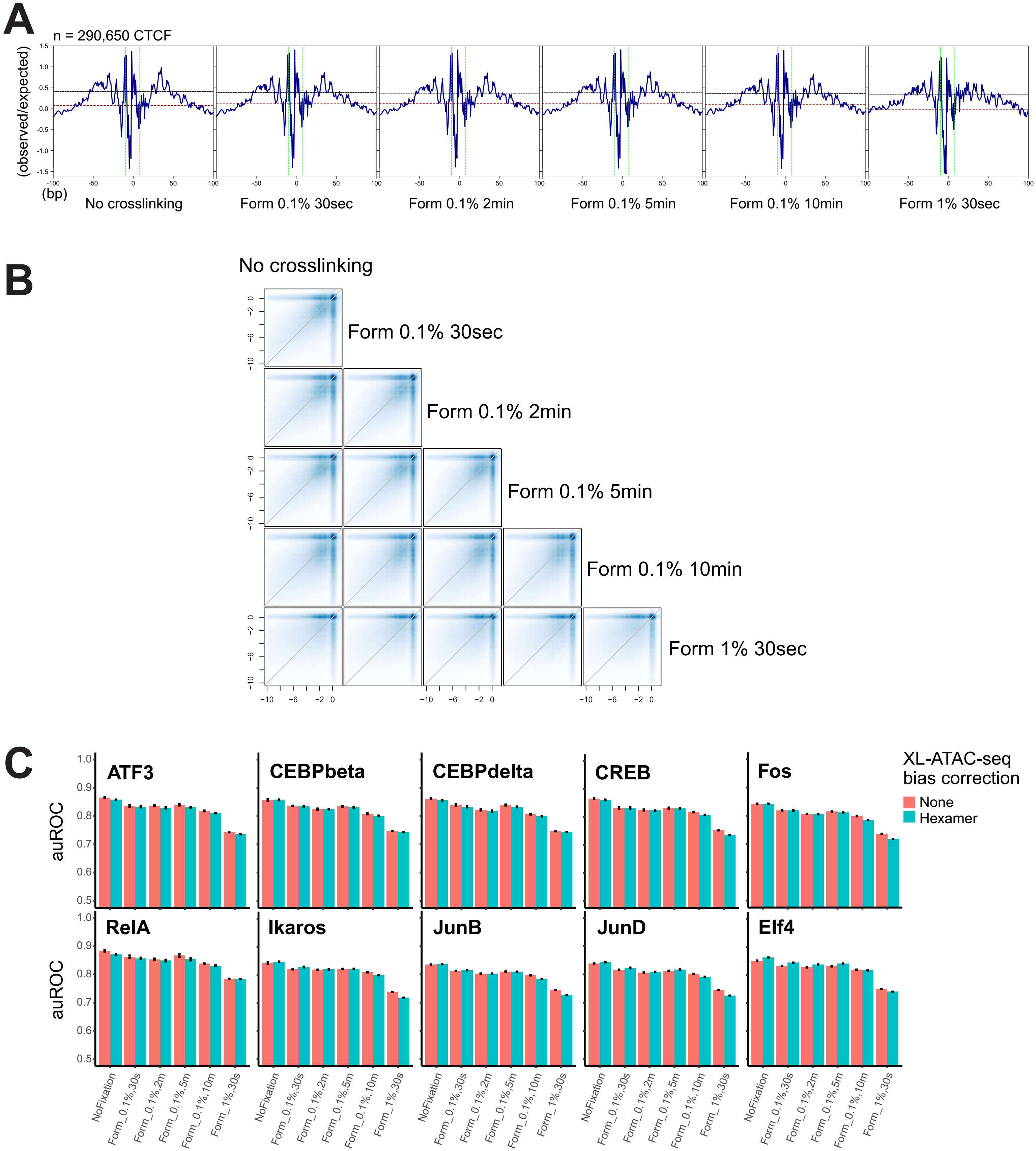

Figure S6. TF footprinting with XL-ATAC-seq (related to Figure 4).
(A) ATAC-seq average insertion count logratio profiles at RelA motif sites. The green vertical dashed lines show the span of the motif. The black horizontal lines indicate flanking cut levels; the red dashed lines mark the average cut levels at the motif. n, total number of motif sites. (B) Comparison of FDR 1% CTCF footprint Z scores across the samples. CTCF motif sites within DHSs (see Methods) across the crosslinking conditions. (C) For TFs with available macrophage ChIP-seq data, area under the ROC curve was calculated in five rounds of subsampling the raw data. Footprints were called by DNase2TF after correcting the insertion count data for DNA sequence bias of Tn5 as indicated. Error bars, s.d.

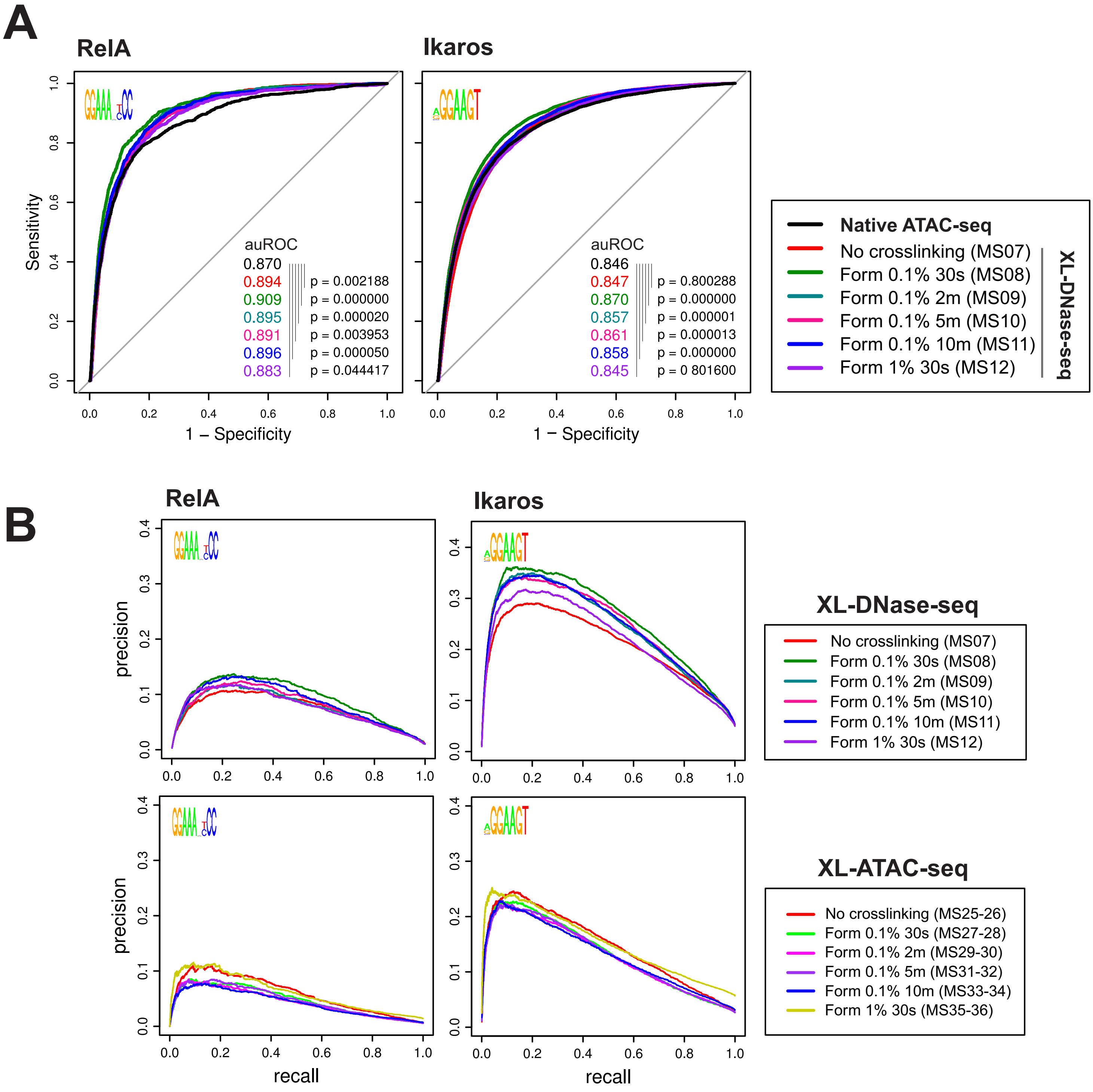

Figure S7. Comparison of TF binding predictions from XL-DNase-seq and XL-ATAC-seq (related to Figures 3-4). (A) The ROC curves in Figure 3 are shown together with the ROC curves from the corresponding native ATAC-seq. The statistical significance of difference between ATAC-seq and the others is represented by the p values. (B) The precision-recall curves correspond to the ROC curves in Figures 3 and 4.

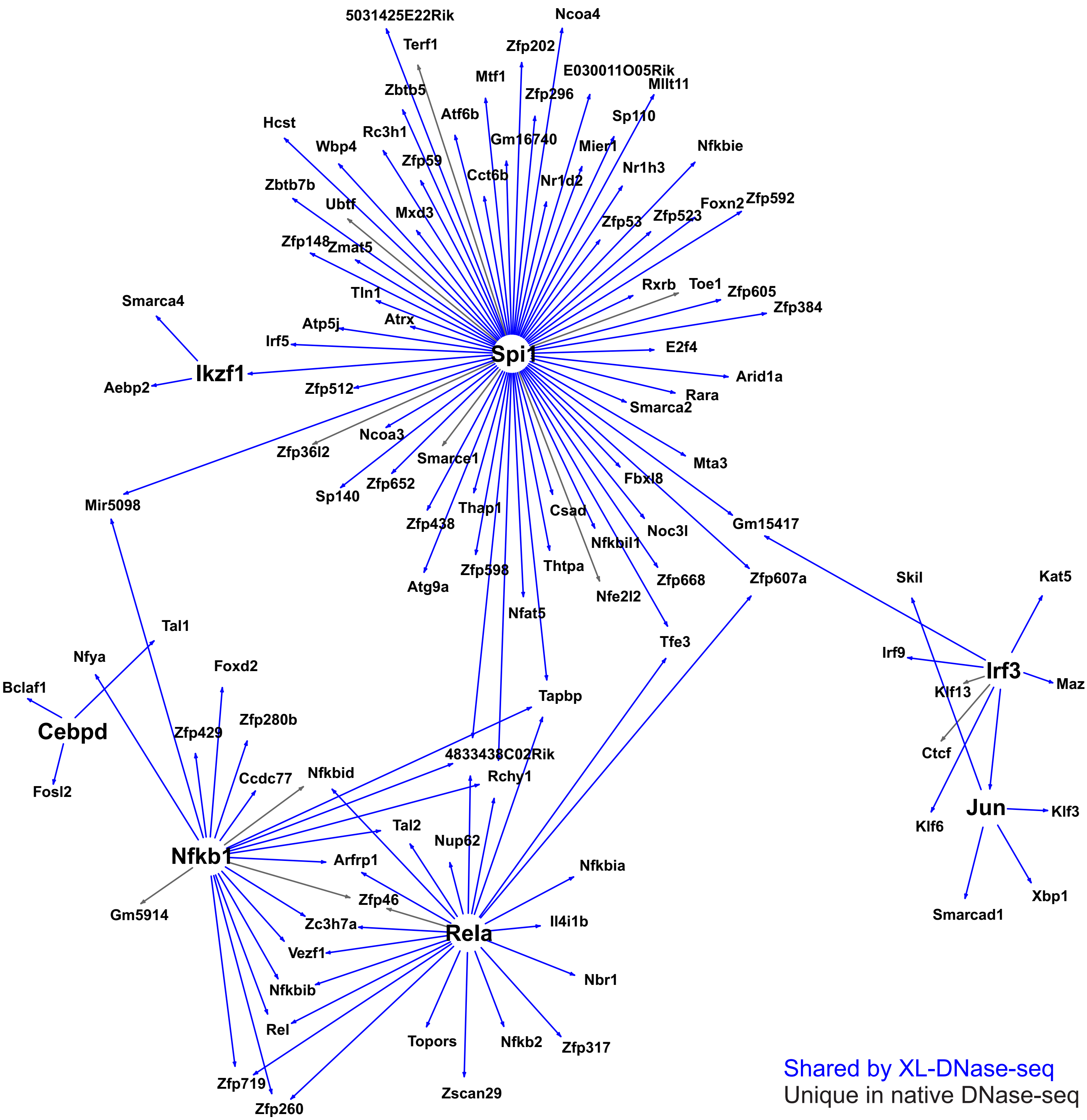

Figure S8. TF regulatory networks constructed by footprints from native DNase-seq (related to Figure 5). Biological replicates of native DNase-seq were pooled and DNase2TF was used to detected footprints at FDR1% after correcting the cut count data for dimer biases. The regulatory connections which are also detected in the networks from any of the XL-DNase-seq samples are marked in blue.

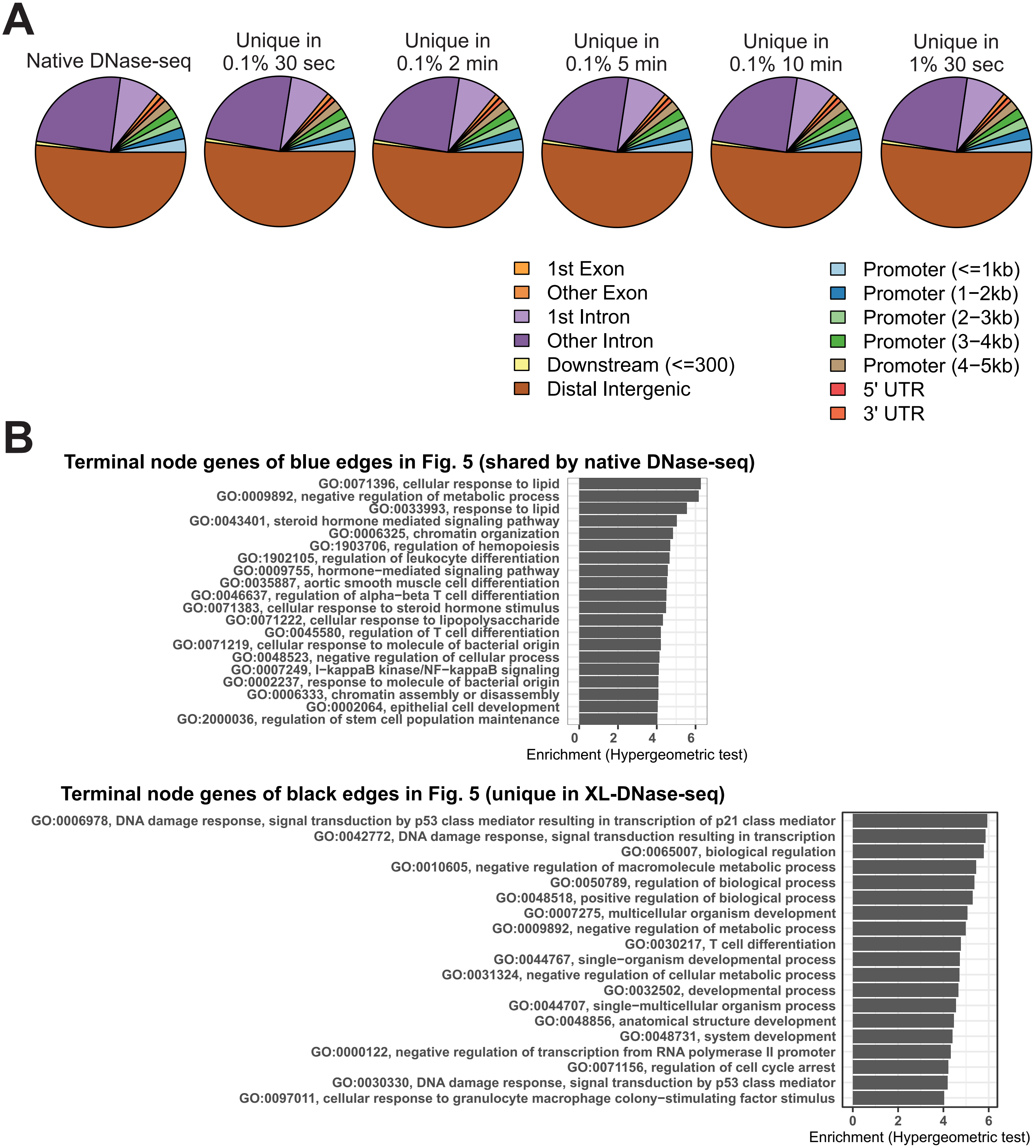

Figure S9. Genomic features and ontology analysis of footprints from XL-DNase-seq (related to Figures 3 and 5).
(A) Genomic distribution of newly detected TF footprints in replicate 2 of XL-DNase-seq samples. Replicate 1 samples produced nearly identical distributions. (B) Gene ontology analysis of genes uniquely containing TF footprints from XL-DNase-seq samples in Figure 5. The expected GO categories from TF genes related to transcriptional regulation were removed from the top to show the other enriched categories.
